# *Lactobacillus rhamnosus* PL1 and *Lactobacillus plantarum* PM1 versus Placebo as Prophylaxis for Recurrence of Urinary Tract Infections in Children

**DOI:** 10.3390/microorganisms12061037

**Published:** 2024-05-21

**Authors:** Maria Daniel, Hanna Szymanik-Grzelak, Janusz Sierdziński, Małgorzata Pańczyk-Tomaszewska

**Affiliations:** 1 Department of Pediatrics and Nephrology, Medical University of Warsaw, 02-091 Warsaw, Poland; maria.daniel@wum.edu.pl (M.D.); malgorzata.panczyk-tomaszewska@wum.edu.pl (M.P.-T.); 2 Department of Medical Informatics and Telemedicine, Medical University of Warsaw, 02-091 Warsaw, Poland; janusz.sierdzinski@wum.edu.pl

**Keywords:** probiotics, prophylaxis, urinary tract infection, *Lactobacillus*

## Abstract

Urinary tract infections (UTIs) rank among the most prevalent bacterial infections in children. Probiotics appear to reduce the risk of recurrence of UTIs. This study aimed to evaluate whether probiotics containing *Lactobacillus rhamnosus* PL1 and *Lactobacillus plantarum* PM1 therapy prevent UTIs in the pediatric population compared to a placebo. A superiority, double-blind, randomized, controlled trial was conducted. In total, 54 children aged 3–18 years with recurrent UTIs or ≥one acute pyelonephritis and ≥one risk factor of recurrence of UTIs were randomly assigned (27 patients in each arm) to a 90-day probiotic or placebo arm. The age, sex, diagnosis, renal function, risk factors, and etiology of UTIs did not vary between the groups. During the intervention, 26% of children taking the probiotic had episodes of UTI, and it was not significantly less than in the placebo group. The number of UTI episodes during the intervention and the follow-up period decreased significantly in both groups, but the difference between them was insignificant. We observed a decrease in UTIs during the study of almost 50% in the probiotic group compared to the placebo group. Probiotics can be used as natural, safe prophylaxis for children with risk factors for UTIs in whom antibiotic prevention is not indicated.

## 1. Introduction

Urinary tract infections (UTIs) are among the most frequently occurring bacterial infections in the pediatric population. The occurrence of UTIs with clinical symptoms in children under the age of 7 is approximated to be 3–7% in girls and 1–2% in boys. It is reported that 8% to 30% of these children experience one or more recurrences of UTIs [1,2,3].

There have been no clear recommendations for the prophylaxis of UTIs in previously published European and global guidelines. Based on current recommendations, including those from the Polish Society of Pediatric Nephrology, children with congenital anomalies of the kidney and urinary tract (CAKUT) who have a history of UTIs and vesicoureteral reflux (VUR) grade ≥3 should be considered for antibacterial prophylaxis [1,2,4,5,6,7].

A few randomized studies have not shown a significant benefit of antibiotic prophylaxis in reducing the frequency of UTIs or preventing renal scarring. Moreover, antibiotic prophylaxis was connected with the development of bacterial resistance [3,8,9].

Lately, there has been an increasing interest in alternative methods for preventing UTIs, like immunotherapy, cranberry, and probiotics [10,11,12,13,14,15]. Using some probiotics appears to reduce the risk of UTIs [16,17,18,19,20]. Lactobacilli strains were used as prophylaxis for the recurrence of UTIs (rUTIs). In 2017, de Llano et al. conducted an in vitro study, showing the initial evidence of *Lactobacillus* strains’ ability to prevent adhesion to bladder epithelial cells by uropathogens [21]. This finding implies that such an action could contribute to the possible advantages of probiotics against UTIs. Due to their inherent ability to migrate along the gastrointestinal tract to the rectum and anus, lactobacilli bacteria can then move to the urethra and vagina [22,23,24]. This migration potential suggests that lactic acid-producing bacteria might positively influence the urogenital microflora by effectively adhering to the epithelial urogenital tracts and displacing uropathogenic microorganisms [15,24,25]. Adhesion can be impeded through exclusion, where lactobacilli occupy binding sites, preventing the initial binding of uropathogens. Additionally, competition occurs as lactobacilli vie with uropathogens for available adhesion receptors on epithelial cells. Moreover, displacement arises when lactobacilli dislodge uropathogens that have already adhered to epithelial cells. Furthermore, the translocation of *Lactobacillus* from the intestinal mucosa to the distal mucosal surfaces is a physiological process that may also explain, at least in part, the colonization of the urogenital tract after oral administration of selected *Lactobacillus* strains [26]. Coaggregation of lactobacilli with uropathogens creates a microenvironment where the antimicrobial compounds produced by lactobacilli (e.g., hydrogen peroxide, lactic acid, and bacteriocin) are localized close to the uropathogens. This localization leads to the suppression of bacterial biofilm formation and a reduction in proinflammatory cytokines (e.g., tumor necrosis factor, interleukin-6, interleukin-8, and interleukin-10) [15,21,22,23,27].

*Lactobacillus* spp. are thought to potentially translocate through the bloodstream as well. In healthy individuals, bacteria are typically captured and eliminated in the mesenteric lymph nodes. Immunocompromised patients, such as those with conditions like cancer or HIV, may experience a weakening of this protective mechanism. This can lead to adverse effects, such as septicemia, endocarditis, bacteremia, and potentially even death [25,28]. In some studies in which probiotics were administered in high doses to healthy individuals, it was observed that there was no translocation of probiotics [29]. Indeed, probiotics rarely lead to severe illness in healthy individuals, even in cases where probiotic bacteria move from the gastrointestinal tract [25,28].

Moreover, lactobacilli have demonstrated antimicrobial efficacy against ESBL-producing *E. coli* and multidrug-resistant pathogens, with the most significant impact observed in strains of *L. plantarum* and *L. fermentum* [30,31]. An explanation for this observation is that lactic acid enhances the permeability of the Gram-negative outer cell membrane to antimicrobial agents (such as hydrogen peroxide), thus increasing their bactericidal effects [31,32]. Lactobacilli have also been found to play a role in regulating the host immune system and preventing infections by immunomodulation [25,33]. Lactobacilli can stimulate the Toll-like receptor pathway, triggering the production of interleukins and myeloid differentiation factor 88, thus initiating an immune response against uropathogens [10]. *L. rhamnosus* GR-1 has been demonstrated to specifically boost the nuclear factor-κB pathway, which is activated by uropathogenic *E. coli*, by releasing various immunomodulatory proteins, such as NLP/P60, GroEL, and elongation factor Tu [34]. The specific impact of these diverse antimicrobial properties on overall clinical effectiveness remains uncertain, as not all *Lactobacillus* strains possess all of these effects. In an in vitro study evaluating 15 different *Lactobacillus* strains, it was observed that *L. crispatus* exhibited a superior capability to inhibit uropathogen adherence to vaginal epithelial cells compared to the other lactobacilli studied. In contrast, other strains (such as *L. jensenii*) were found to possess a greater capacity to directly inhibit the growth of uropathogens [23]. Another study showed that only 82% of the total *Lactobacillus* strains investigated could produce hydrogen peroxide, and merely 68% could produce bacteriocin [35]. Lee et al. carried out a randomized trial involving children with persistent primary VUR and rUTIs [36] and children under one-year-old with VUR who had experienced pyelonephritis [17]. The study aimed to compare the efficacy of *L. acidophilus* with low-dose trimethoprim and sulfamethoxazole therapy (TMP/SMX). No significant difference was observed in the impact of probiotic and antibiotic prophylaxis on rUTIs [17,36]. Some intervention studies have reported whether administering specific *Lactobacillus* strains can prevent UTIs [37,38]. The most recent research showed diversity in the methodologies employed [14,16,18,19,20].

The effects of probiotics are strain-specific, and these heterogeneities are crucial when evaluating their potential efficacy as therapeutic agents for preventing UTIs. In this paper, the investigators aim to assess the impact and safety of administering probiotics containing *L. rhamnosus* PL1 with *L. plantarum* PM1 in preventing rUTIs in children.

## 2. Materials and Methods

### 2.1. Trial Objectives and Hypothesis

The effects of probiotics are strain-specific. The primary aim of this study is to assess the efficacy and safety of probiotics containing *Lactobacillus rhamnosus* PL1 and *Lactobacillus plantarum* PM1 in preventing UTIs in children compared to a placebo.

We hypothesize that the study product is more effective than a placebo in the prophylaxis of UTIs in children. The trial is registered at ClinicalTrials.gov (NCT03462160). The trial protocol and methodology have been previously published [39]; thus, we are providing a briefer version.

### 2.2. Trial Design

This study is designed as a randomized, placebo-controlled, double-blind, superiority trial.

### 2.3. Settings and Participants

The study was performed in pediatric units of the pediatric hospital and the nephrology outpatient clinic of the Medical University of Warsaw. The recruitment started in July 2018; the last patient was recruited in January 2022.

### 2.4. Eligibility Criteria

Participants had to meet all the following inclusion criteria to be recruited for the trial:

- aged from 3 to 18 years;
- diagnosis of recurrent UTIs in the last year, defined as [7]:
  - ≥2 episodes of acute pyelonephritis (APN)/upper UTI;
  - 1 episode of APN and ≥1 episode of cystitis/lower UTI;
  - ≥3 episodes of cystitis/lower UTI;

or 1 episode of APN and ≥1 of UTIs risk factors: CAKUT, constipation, bladder dysfunction, neurogenic bladder, hypercalciuria, and sexual activity in girls;

- ≥1 episode of UTI in the last 6 months.

Participants provided written informed consent before enrolment.

### 2.5. Exclusion Criteria

Exclusion criteria included the intake of probiotic preparations for ≥1 month in the last 3 months; antibiotic use within the previous month due to any reason; a known allergy to the study products; immunosuppression therapy; a disease with immune deficiency; a central catheter; and children with severe coexisting infections, e.g., meningitis, sepsis, pneumonia, and otitis.

### 2.6. Interventions

**Figure 1:**
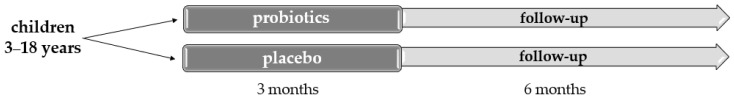
The study period was divided into 3-month interventions and 6 months of follow-up.

Participants were enrolled for a 9-month study period, which included 12 weeks of treatment (Figure 1).

Oral and written information was given to each participant’s parents and children >16 years old. Participants were randomized during hospitalization or visits to outpatient clinics and required to take a study product or placebo orally. Eligible patients received *L. rhamnosus* PL1 with *L. plantarum* PM1 at a dose of 10^9^ CFU (2 g) each or a placebo, orally, once daily, in the evening during a meal, after dissolving the powder in lukewarm water. The probiotics or placebo were administered for 90 days. Throughout the study period, caregivers recorded the UTIs. Caregivers had the right to withdraw a participating child from the study at any time, and they were not obliged to give reasons for this decision, which did not affect subsequent medical care. In the event of UTIs, the proper treatment was implemented according to Polish guidelines [7].

Furthermore, all patients and their caregivers were educated on proper toileting and hygiene practices. Patients with risk factors for UTIs were adequately treated.

All participants were provided probiotics containing *L. rhamnosus* PL1 (B/00055) with *L. plantarum* PM1 (PCM2572) or a placebo. The probiotics powder also contained excipients (potato maltodextrin, glucose, gum arabic, pectin, and silicon dioxide). The placebo formulation was identical to the active products but without probiotic bacteria. The combination of *L. rhamnosus* PL1 and *L. plantarum* PM1 was based on their strengths in gastrointestinal and urogenital colonization and immunomodulation and their potential synergistic effects in preventing UTIs. The placebo’s appearance closely resembled that of the probiotic-containing powder. Using a placebo is the gold standard for evaluating new treatment efficacy, so it was selected as the comparator in our trial. The study products (the probiotics and placebo) were manufactured and supplied by Miralex (Pila, Poland) free of charge. The manufacturer did not participate in the conception and protocol preparation, design, and conduct of the study or in analyzing and interpreting the data. 

### 2.7. Follow-Up

All study participants were followed up directly after the intervention at 3 and 6 months.

### 2.8. Compliance

Face-to-face interview with patients and/or caregivers and through a daily patient diary (prepared by researchers and returned upon intervention completion) was conducted to assess compliance with the study. Based on previously published trials [12,17,36], participants receiving less than 75% of the recommended doses were deemed non-compliant.

The study period was during the COVID-19 pandemic, so due to the isolation period, patients did not return the sachets (either empty or those they had left over) as was planned in the study protocol [39].

### 2.9. Outcome Measures

The primary outcome measure was the number of episodes of UTIs during the intervention and 6 months after the intervention. The secondary outcome measures were the number of days of hospitalization due to UTI and the number of days of antibiotic therapy due to UTI.

### 2.10. Sample Size/Sequence Generation

A power and sample-size calculator for the binary outcome superiority trial was used to estimate the study and control groups. Due to the COVID-19 pandemic and difficulties recruiting patients during this period, the study was terminated before the planned number of children was included. 

An independent researcher affiliated with the Medical University of Warsaw generated the randomization list. Block randomization with a block size of 6 was used. Researchers and participants were not assigned to the patient group during the study. Randomization codes were revealed when all data were collected, and the final analysis was performed.

### 2.11. Allocation Concealment

Allocation concealment was processed using opaque, sealed, and numbered envelopes. It was implemented after getting informed consent and entering essential demographic information into the case report form (CRF). A randomization list was generated by a computer that assigned an independent person the numbered study products.

### 2.12. Blinding

The probiotic and placebo were packaged in identical sachets. The placebo powder looked and tasted similar. The sachets were delivered by Miralex in sealed and sequentially numbered opaque envelopes. The intervention was blinded for all participants and investigators until the end of the study.

### 2.13. Data collection and Management

All participants were ensured data confidentiality during the workshop process. All study participants were allocated a study identification number. The data were collected and stored in an electronic database protected by a password. Only the researchers involved had access to all participant records, CRFs, documents, laboratory data (serum creatinine level), etc.

### 2.14. Statistical Analysis

An intention-to-treat (ITT) analysis was performed, and all randomly assigned participants whose outcomes were approachable (including dropouts and withdrawals) were included. The ITT analysis on the primary and secondary outcomes was processed. This analysis included all children recruited to the study (children who completed the entire treatment protocol as initially scheduled, with follow-up available 6 months post-intervention, including dropouts). 

Statistical analysis was conducted using Statistica 13.0 software. Categorical data were reported as absolute frequencies and percentages. The normality of continuous data distribution was assessed using the Shapiro–Wilk test. Non-parametric Mann–Whitney U and Wilcoxon tests were used to demonstrate the significance of differences for the study groups. Chi^2^ tests were performed for binary outcome measures. A P-value less than 0.05 was deemed statistically significant for all tests.

### 2.15. Harms

Any adverse events resulting from participation in the trial were registered.

### 2.16. Ethics and Dissemination

The Bioethics Committee of the Medical University of Warsaw reviewed and accepted the study protocol and template consent (KB/6/2018). Verbal and written information about informal consent was revealed to the caregivers. A parent or legal guardian signed the informed consent forms before randomization. Patients could abandon the study at any time without warning, as documented and explained at the time of providing consent.

The full protocol is freely due to open-access publication [39].

## 3. Results

**Figure 2:**
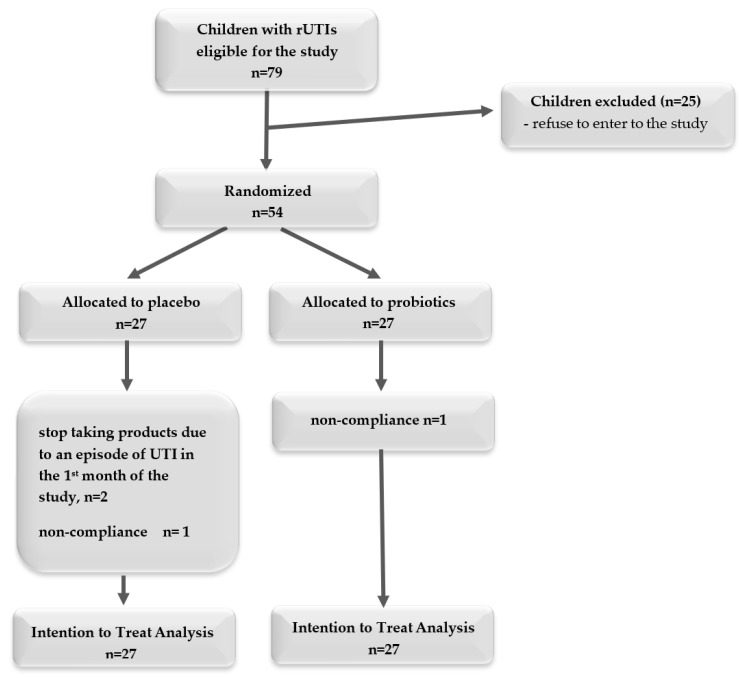
A flowchart of the recruiting process.

We randomized 54 children, with half allocated to the placebo and half to the probiotics arm. Four children did not finish the intervention, but we conducted an ITT analysis (Figure 2). Among the children who received the probiotic, one child did not take the product because it was untasted. Three children who received a placebo did not complete the study: two had UTIs in the first month of the study, and parents decided to discontinue taking the product, and one took the product irregularly (<75% of recommended doses).

The study groups were comparable in age, sex, diagnosis, renal function, risk factors, and etiology of UTIs. In the study group, the median age was 8.5 years (IQR 5.1–11 years), with normal renal function (based on eGFR according to the Schwartz formula), and the majority of children were girls (92.6%). In the year before the study, 48.1% of patients were diagnosed with APN and cystitis, 42.6% had only cystitis, the others were diagnosed with APN (9.3%), and *E. coli* caused UTIs in 72.2% of cases. In total, 92.6% of patients had at least one risk factor for UTI; in both the probiotic and placebo groups, the most common risk factors were bladder dysfunction and constipation (77.8% vs. 77.8% and 51.8% vs. 33.3%, respectively) (Table 1). 

None of the patients received any prophylaxis for UTI. Some study participants were reluctant to give up yogurt for the duration of the study. 

During the intervention, 26% of children taking the probiotic had episodes of UTI, which is less than in the placebo group (44.4%), but this difference was not significant (P = 0.154). A similar number of children had UTIs during the follow-up period (Table 2).

**Figure 3:**
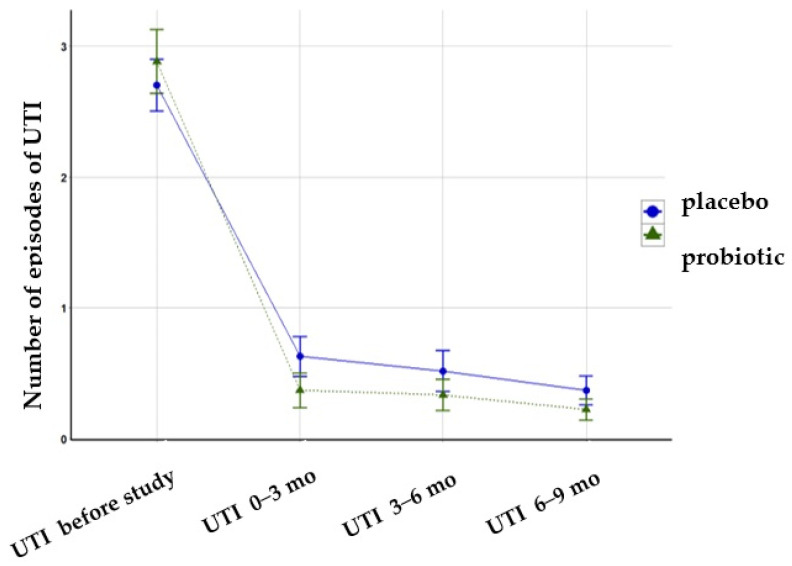
The number of UTI episodes during the intervention and follow-up period.

Before the study, the number of episodes of UTI was comparable in both groups (placebo 2.7 vs. probiotic group 2.88, P = 0.656). The number of UTI episodes during the intervention and the follow-up period decreased significantly in both groups, but the difference between them was insignificant (Figure 3).

**Figure 4:**
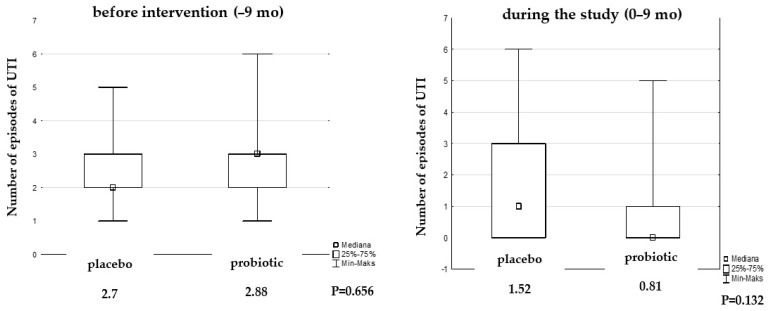
Median of UTI episodes before and during the study.

We observed a decrease in the number of episodes of UTI during the study by almost 50% in the probiotic group compared to the placebo group (0.81 vs. 1.52 episodes, Figure 4).

**Figure 5:**
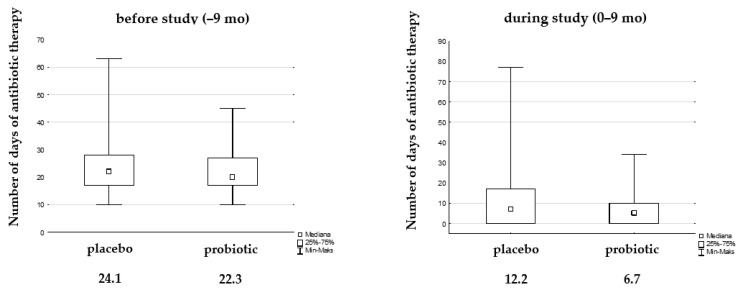
Median of days antibiotic therapy due to UTIs before and during the study.

The number of days of antibiotic therapy during the study decreased statistically significantly in each group compared to the 9 months before the study, while the difference between them was not significant. However, we observed a reduction in days of antibiotic therapy by almost 50% in the probiotic group (12.2 vs. 6.7 days, Figure 5).

The number of days hospitalized for UTIs during the study also decreased significantly in the placebo and probiotic groups, without any difference between them: before the study, the placebo vs. probiotic groups were 6.11 vs. 6.59 (P = 0.836), and during the study, they were 1.7 vs. 1.3 (P = 0.849).

There were no adverse events, although some children did not tolerate the chalky lemon taste.

## 4. Discussion

Only several clinical trials have explored the efficacy of different probiotics in preventing UTIs in children.

In our randomized, controlled trial (RCT), we found that probiotics containing *L. rhamnosus* PL1 and *L. plantarum* PM1 decreased the number of children with UTIs during the intervention and the follow-up period significantly in both groups, but the difference between them was not significant. Probiotics reduced episodes of UTI mainly during the intervention period. 

In a small retrospective study, Madden-Fuentes et al. investigated the efficacy of a combination therapy of fluoroquinolone for 14 days and a probiotic (*Saccharomyces boulardii*) for one year in children with rUTIs. The study revealed a significant reduction in UTI episodes, with seven out of ten children remaining free of UTIs during the follow-up period, which ranged from 3 to 15 months [40].

Lee et al. conducted a comparison between the effectiveness of probiotics (*L. acidophilus* Antibio300^®^, 1×10^8^ CFU/g bid, Hanwha Co, Seoul, Korea. or *L. acidophilus* and *L. rhamnosus*—Lacidofil^®^, 2×10^9^ CFU/g bid, Phambio Co., Seoul, Korea) prophylaxis for rUTIs in infants with APN with antibiotic therapy or those without prophylaxis. The study showed that only 8.2% of infants experienced rUTIs during a 6-month follow-up period [11]. However, probiotic treatment demonstrated greater efficacy than no prophylaxis, and its effectiveness was on par with antibiotic treatment. Sadeghi-Bojd et al. found that most children without CAKUT who received treatment with a probiotic formulation including 11 diverse strains of probiotics (Complete Probiotic Platinum 1MD, Sherman Oaks, Los Angeles, CA, USA: *L. acidophilus* 15 × 10^9^ CFU), *L. rhamnosus* 1×10^9^ CFU, B. bifidum 4 × 10^9^CFU, and *B. lactis* 15×10^9^ CFU) successfully recovered from their first febrile UTI and remained free from rUTI for 18 months [37]. In children with persistent primary VUR, Lee et al. compared the effectiveness of *L. acidophilus* probiotic (Antibio300^®^, Hanwha Co., Seoul, Korea, 1.0 × 10^8^ CFU/g ATCC 4356) in preventing rUTIs to antibiotic treatment (TMP/SMX) [36]. It has been established that both treatments effectively prevented rUTIs in these patients.

Mohseni et al. conducted a comparison between the effectiveness of a combined therapy involving probiotics *L. acidophilus* (LA5) and *B. lactis* (BB12) and nitrofurantoin in preventing rUTIs in children with unilateral VUR and rUTIs [12]. The combined treatment decreased the occurrence of rUTIs among treated patients. No statistically significant difference was observed between groups.

Meena et al., in a meta-analysis of RCTs, showed that probiotics were more effective than a placebo and comparable to antibiotic treatment in children with rUTIs [19]. Hosseini et al. found in a meta-analysis that combining probiotic therapy with antibiotics effectively prevents rUTIs in children [41]. Emami et al., based on eleven systematic reviews and meta-analyses, found that probiotics may be an alternative natural prophylaxis for UTIs in children [20]. Hudson et al. concluded that *Lactobacillus* strains seem to be the most effective in prophylaxis recurrent UTIs in pregnant women and children [42].

Meanwhile, a Cochrane review of nine RCTs observed that probiotics do not show a benefit over a placebo in reducing rUTIs [18].

In our trial, we noted that multifactorial effects may have impacted reducing rUTIs. In our group, 92.6% of children had at least one risk factor for UTI. Bowel and bladder dysfunction (BBD) was diagnosed in over 77% of patients in the study group and was adequately treated. Many authors observed a positive correlation of BBD and rUTI [43,44,45,46]. Axelgaard et al. found that constipation was positively associated with rUTI in children, particularly girls aged 4 to 18 years without CAKUT [47]. In our study group, we observed a similar dependence.

The observed decrease in UTIs in the probiotic group of almost 50% compared to the placebo group may indeed be attributed to the improvement of gut microbiota and colonization of the perineum with *Lactobacillus* bacteria. However, further confirmation would require stool culture samples to be collected before and after the intervention. Additionally, factors such as enhanced immune responses due to probiotic supplementation, the modulation of inflammatory pathways, and the competitive exclusion of uropathogens by the probiotic strains could have contributed to the lower incidence of UTI in children receiving *L. rhamnosus* PL1 and *L. plantarum* PM1 compared to those on the placebo. 

We acknowledge certain limitations in our study that may account for the lack of significant difference observed between the probiotic and placebo groups. These limitations primarily stem from the relatively small sample size in the group, comprising 54 children, which was influenced by the challenges of patient recruitment during the COVID-19 pandemic. Another limitation of our research could be the relatively short duration of observation (9 months). 

In our trial, we also observed that the number of days of hospitalization for UTIs during the study decreased significantly in the placebo and probiotic groups without a difference between them. This is important not only for patients but also from a socioeconomic point of view. More than 1 million annual physician office visits, 500,000 emergency department visits, and more than 50,000 hospital admissions are attributed to children diagnosed with UTIs in the USA. The number of inpatient and outpatient visits related to the treatment of ZUM has been steadily increasing since 2000 [48,49]. To reduce the growing number of UTI clinical encounters and the rising costs related to hospitalization, it is imperative for physicians to promptly identify, assess, and treat UTIs adequately, as well as effectively prevent UTI recurrences.

## 5. Conclusions

Based on our trial, systematic review, and meta-analysis, probiotics might be considered an alternative prophylactic therapy for children with rUTIs. Probiotics can be used as natural and safe prophylaxis for children with risk factors for UTIs in whom antibiotic prevention is not indicated.

However, the current published studies show high heterogeneity, emphasizing the need for further large, randomized trials employing well-characterized *Lactobacillus* strains. Strengthening the quality of evidence for probiotics requires larger and more robust trials.

## Figures and Tables

**Table 1:** Characteristics of the study group.

	All Study Groups	Placebo	Probiotic	Placebo vs. Probiotic
Age, years (median, IQR)	8.5 (5.8–11)	9.1 (5.8–11.2)	8.4 (5.4–11.1)	P = 1
Gender (F/M)	50/4	25/2	25/2	P = 1
**Diagnosis** (*n*, %)				
- APN	5 (9.3%)	3 (11.1%)	2 (7.4%)	P = 0.656
- Cystitis	23 (42.6%)	11 (40.8%)	12 (44.5%)	P = 0.988
- APN + Cystitis	26 (48.1%)	13 (48.1%)	13 (48.1%)	P = 1
eGFR (ml/min/1.73 m^2^) (median, IQR)	118.7 (105.3–139)	115.6 (104.8–136.3)	119 (108.4–146.6)	P = 0.612
**Risk factors of UTI** (*n*, %)	50 (92.6%)	24 (88.9%)	26 (96.3%)	P = 0.586
- bladder dysfunction (*n*, %)	42 (77.8%)	21 (77.8%)	21 (77.8%)	P = 1
- constipation (*n*, %)	32 (59.2%)	14 (51.8%)	18 (33.3%)	P = 0.268
- CAKUT (*n*, %)	14 (25.9%)	8 (14.8%)	6 (22.2%)	P = 0.536
- neurogenic bladder (*n*, %)	4 (7.4%)	1 (3.7%)	3 (11.1%)	P = 0.299
- hypercalciuria (*n*, %)	5 (9.2%)	3 (11.1%)	2 (7.4%)	P = 0.656
- sexual activity (*n*, %)	2 (3.7%)	1 (3.7%)	1 (3.7%)	P = 1
**UTI etiology** (*n*, %)				
- *Escherichia coli*	39 (72.2%)	20 (74.1%)	19 (70.4%)	P = 0.457
- *Escherichia coli* + others	13 (24%)	5 (18.5%)	8 (29.6%)	P = 0.426
- *Enterococcus* sp.	1 (1.9%)	1 (3.7%)	0 (0%)	
- *Klebsiella* sp.	1 (1.9%)	1 (3.7%)	0 (0%)	

F—female, M—male, APN—acute pyelonephritis, eGFR—estimated glomerular filtration according to Schwartz formula, UTI—urinary tract infection, CAKUT—congenital anomalies of kidney and urinary tract.

**Table 2:** The number of children with UTIs during the intervention (0–3 months), follow-up periods (3–9 months), and whole study (0–9 months).

	% Children with UTI		
	Placebo	Probiotic	Placebo vs. Probiotic
0–3 months (*n*, %)	12 (44.4%)	7 (25.9%)	P = 0.54
3–9 months (*n*, %)	11 (40.7%)	9 (33.3%)	P = 0.573
0–9 months (*n*, %)	12 (55.6%)	14 (44.2%)	P = 0.576

## Data Availability

The data presented in this study are available on request from the corresponding author.
